# Analysis of Acetabular Cup Positioning and Functional Outcomes in Total Hip Replacement

**DOI:** 10.7759/cureus.88569

**Published:** 2025-07-23

**Authors:** Jaiveer Singh, Krishan Kumar, Rohan Krishnan

**Affiliations:** 1 Orthopaedics, ESIC-PGIMSR (Employees' State Insurance Corporation Hospital and Postgraduate Institute of Medical Sciences and Research) Basaidarapur, New Delhi, IND; 2 Orthopaedics and Sports Medicine, ESIC (Employees' State Insurance Corporation) Medical College &amp; Hospital, Faridabad, IND

**Keywords:** acetabular cup, anteversion, harris hip score, inclination, total hip replacement

## Abstract

Introduction: Acetabular cup positioning is critical in total hip replacement (THR) for achieving optimal joint biomechanics and preventing complications. This study evaluates the relationship between postoperative cup orientation, measured via computed tomography (CT), and functional outcomes assessed using the Harris Hip Score (HHS).

Methods: A prospective observational study was conducted that included 30 patients undergoing primary THR. Cup inclination and anteversion were measured postoperatively via CT scans. Functional evaluation was done using HHS and range of motion (ROM) at six months. Statistical analysis included paired t-tests and descriptive metrics.

Results: The mean anteversion was 15.3° ± 5.85°, and the mean inclination was 42.7° ± 5.31°. Significant ROM improvements were observed (p<0.001), but no significant correlation was found between cup orientation and HHS (anteversion: r=0.12, p=0.52; inclination: r=0.08, p=0.67).

Conclusion: We conclude that precise cup positioning enhances stability, but functional outcomes depend on multiple factors, including comorbidities and rehabilitation. CT evaluation post-surgery offers a reliable assessment tool. Larger, multicenter studies with extended follow-up are warranted to confirm these results.

## Introduction

Total hip replacement (THR) is a widely performed surgical procedure aimed at alleviating pain and restoring function in patients with severe hip joint pathology, such as avascular necrosis (AVN), osteoarthritis, and femoral neck fractures [1,2]. The success of THR depends on multiple factors, including implant design, surgical technique, and precise positioning of the acetabular and femoral components [3,4]. Among these, acetabular cup positioning is critical, as it directly influences joint biomechanics, stability, and long-term implant survivorship [5,6]. Malpositioning of the acetabular cup can lead to complications, such as dislocation, impingement, accelerated polyethylene wear, and reduced range of motion (ROM), significantly impacting patient outcomes [7,8]. Notably, studies have identified factors such as excessive anteversion and inclination as key contributors to dislocation risk, emphasizing the need for precise alignment [9].

The Lewinnek safe zone (anteversion 5°-25°, inclination 30°-50°) has long served as the benchmark for optimal acetabular cup orientation, with studies demonstrating reduced dislocation rates when cups are positioned within these parameters [5]. However, achieving precise alignment intraoperatively remains challenging, particularly when relying on anatomical landmarks without advanced navigation tools [10]. Postoperative computed tomography (CT) scans offer a reliable method to validate cup orientation, providing accurate measurements of anteversion and inclination compared to traditional radiographs [11]. Despite its importance, the relationship between cup positioning and functional outcomes, such as those measured by the Harris Hip Score (HHS), a widely used metric for assessing hip function post-THR [12], is not fully elucidated, with some studies reporting conflicting findings on whether strict adherence to the safe zone correlates with improved patient-reported outcomes [13].

## Materials and methods

Study design and setting

This prospective observational study was conducted at the Department of Orthopaedics, ESIC-PGIMSR (Employees' State Insurance Corporation Hospital and Postgraduate Institute of Medical Sciences and Research), Basaidarapur, New Delhi, India, from January 2018 to December 2020. The study evaluated acetabular cup positioning and functional outcomes in primary total hip replacement (THR), with outcomes assessed using the Harris Hip Score, a validated tool for measuring functional recovery post-THR [14]. Ethical approval was obtained from the Institutional Review Board (ESIPGIMSR-IEC/2018026), and all participants provided written informed consent.

Sample size and patient selection

A sample size of 30 patients was calculated using a power analysis, assuming a correlation coefficient of 0.5 between acetabular cup orientation and HHS, with 80% power and a 5% significance level [15]. Patients aged 18-80 years undergoing primary THR, those diagnosed with avascular necrosis (AVN), secondary osteoarthritis (OA), or femoral neck fracture, and those who were willing to provide informed consent and complete follow-up were included. Exclusion criteria included having revision THR or bilateral THR, severe comorbidities (e.g., uncontrolled diabetes, active malignancy), and incomplete follow-up data or non-compliance with postoperative assessments.

Surgical technique

All THRs were performed by a single surgeon using a posterior approach guided by anatomical landmarks [13]. Cemented or cementless implants (DePuy Synthes, New Brunswick, NJ, USA) were selected based on bone quality, age, and surgeon preference. Implants and investigations were provided free of cost, and no off-label drugs or devices were used.

Data collection

**Figure 1:**
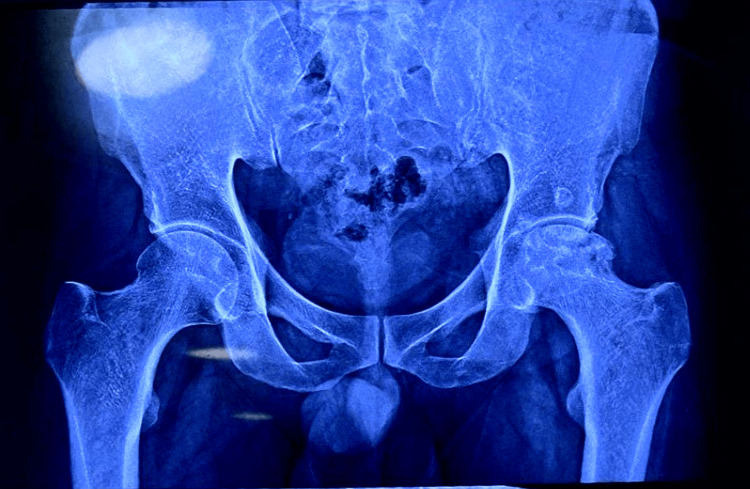
A preoperative X-ray of the pelvis The anteroposterior pelvic radiograph shows advanced degenerative changes in the left hip joint due to avascular necrosis prior to total hip replacement.

**Figure 2:**
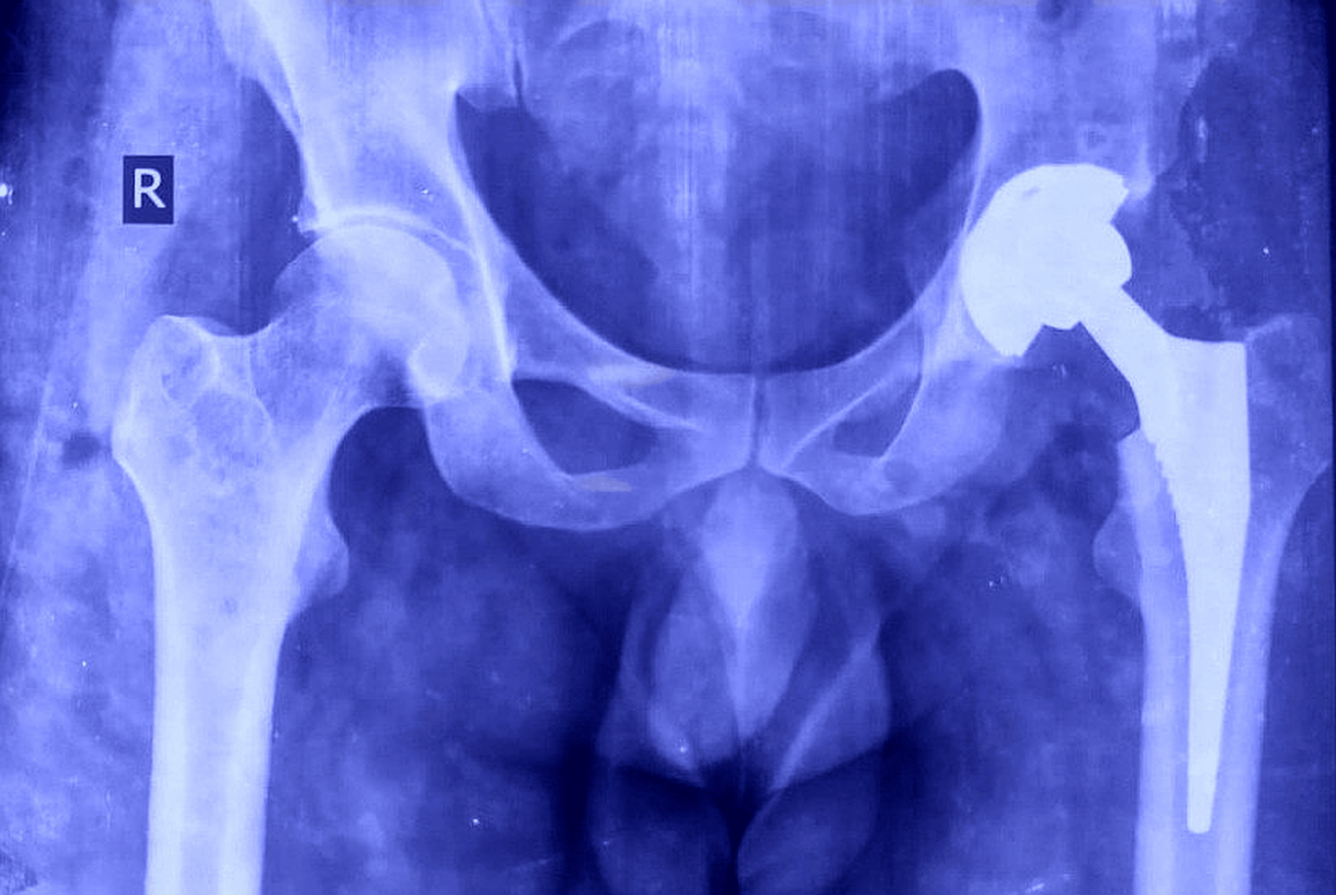
A postoperative X-ray after total hip replacement The anteroposterior radiograph demonstrates proper positioning of the acetabular cup and femoral stem following cementless total hip replacement.

**Figure 3:**
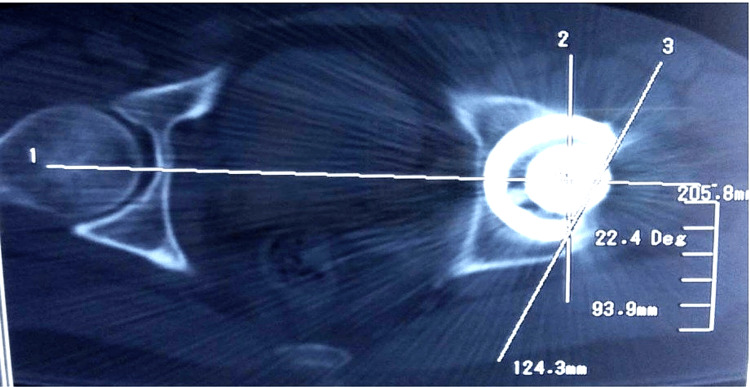
A CT scan showing acetabular anteversion angle measurement The axial CT scan demonstrates the measurement of acetabular cup anteversion using the modified Murray method [13]. Line 1 represents the inter-teardrop line, Line 2 is drawn perpendicular to Line 1 as a reference, and Line 3 indicates the cup opening. The anteversion angle, measured between Line 2 and Line 3, is 22.4°.

**Figure 4:**
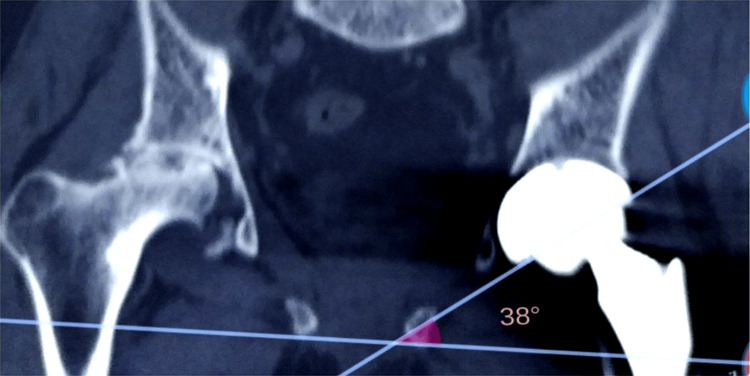
A CT scan showing acetabular inclination angle measurement The coronal CT scan shows the cup inclination angle of 38°. The angle was calculated between the cup opening and the inter-ischial line as per standard CT-based assessment methods.

Preoperative assessments included demographics, clinical indications (AVN, osteoarthritis, femoral neck fracture), and ROM measured via goniometry. Anteroposterior (AP) pelvic radiographs and lateral views of the affected hip confirmed diagnoses (Figure 1). Postoperative anteroposterior and lateral radiographs were used to assess implant positioning (Figure 2). Postoperative CT scans, performed within two weeks, were used to measure cup orientation. For anteversion, the angle between anterior-to-posterior cup margins and a perpendicular to the inter-teardrop line was calculated using the modified Murray method (Figure 3 illustrates the measurement) [13]. For inclination, the measurement was taken relative to the transischial line, as established by prior research (Figure 4) [5]. Measurements were compared to the Lewinnek safe zone (anteversion 5°-25°, inclination 30°-50°). Functional outcomes were assessed at six months using the modified Harris Hip Score [14].

Statistical analysis

Data were analyzed using IBM SPSS Statistics, version 25.0 (IBM Corp., Armonk, NY). Descriptive statistics included means, standard deviations (SDs), and percentages. Normality was confirmed using the Shapiro-Wilk test. Paired t-tests were used to compare preoperative and postoperative ROMs (flexion-extension, abduction-adduction, internal-external rotation). Pearson’s correlation was used to assess associations between cup orientation (anteversion and inclination) and HHS. Significance was defined as p<0.05 (two-tailed tests).

Quality assurance

CT measurements were independently verified by two radiologists, with discrepancies resolved by consensus. To minimize variability, ROM assessments were done by a single physiotherapist. For imaging, a 64-slice CT scanner (GE Healthcare, Chicago, IL, USA) and digital radiography were used. Data entry was double-checked for accuracy.

## Results

Patient characteristics

The study cohort comprised 30 patients, with predominantly male patients (80%, n=24); the mean age was 58.2 ± 12.3 years (range: 32-78). As shown in Table 1, avascular necrosis was the primary indication for THR (60%, n=18), followed by secondary osteoarthritis (23%, n=7) and femoral neck fracture (17%, n=5). This distribution reflects regional trends, where AVN is prevalent due to corticosteroid use and trauma, consistent with prior studies [16]. The predominance of male patients may be attributed to higher rates of traumatic injuries and occupational risk factors in the study population, aligning with epidemiological data on hip pathologies in India [17].

**Table 1:** Patient demographics and indications AVN = avascular necrosis; OA = osteoarthritis

Characteristic	No. of patients (n=30)	Percentage
Male	24	80%
Female	6	20%
AVN	18	60%
Secondary OA	7	23%
Femoral neck fracture	5	17%

Acetabular cup orientation

Acetabular cup orientation was assessed using postoperative CT scans, with results summarized in Table 2. The mean anteversion was 15.3° ± 5.85° (range: -5° to 26°), with 96.67% (n=29) of cups positioned within the Lewinnek safe zone (5°-25°) (Figure 3) [13]. One patient with a retroverted cup (-5°) experienced a posterior dislocation and transient sciatic nerve neuropraxia, necessitating revision THR, a complication often linked to malpositioning as noted in prior classifications of THR dislocations [18]. This complication underscores the critical importance of avoiding retroversion, as it disrupts joint stability and increases impingement risk, a finding consistent with biomechanical studies [5]. The mean inclination was 42.7° ± 5.31° (range: 35°-57°), with 93.33% (n=28) within the safe zone (30°-50°) (Figure 4) [5]. Two patients had inclination angles slightly above the safe zone (51°, 57°), yet neither experienced complications, suggesting that minor deviations in inclination might be less critical than anteversion errors in this cohort [19]. These results highlight the efficacy of anatomical landmark-guided surgery in achieving optimal cup positioning in most cases, though extreme malpositioning, such as retroversion, significantly elevates complication risk, impacting patient safety and surgical success.

**Table 2:** Acetabular cup orientation parameters The safe zone is defined as 5°-25° anteversion and 30°-50° inclination based on the Lewinnek criteria [5].

Parameter	Mean ± SD	Range	Within safe zone (%)
Anteversion (°)	15.3 ± 5.85	-5 to 26	96.67
Inclination (°)	42.7 ± 5.31	35 to 57	93.33

Range of motion improvements

Significant improvements in the range of motion were observed across all measured parameters, as detailed in Table 3. Flexion-extension improved from a preoperative mean of 81.5° ± 18.39° to 108.67° ± 12.17° postoperatively (mean difference 27.17°, p<0.001). Abduction-adduction increased from 24.83° ± 11.48° to 43.17° ± 8.25° (mean difference 18.33°, p<0.001), and internal-external rotation rose from 10.83° ± 10.59° to 45.83° ± 8.52° (mean difference 35.00°, p<0.001). These statistically significant improvements (p<0.001) reflect substantial restoration of hip joint kinematics, enabling patients to perform daily activities such as walking, climbing stairs, and sitting with greater ease. The large mean difference in internal-external rotation (35.00°) is particularly notable, as rotational mobility is critical for functional tasks and reducing compensatory movements that may lead to secondary musculoskeletal issues [13]. These ROM gains suggest that precise cup positioning, combined with effective soft tissue balancing, contributes to biomechanical stability and enhanced patient mobility, aligning with the goals of THR to improve quality of life.

**Table 3:** Comparison of preoperative and postoperative ROM parameters ROM = range of motion p-values from paired t-tests were used to compare preoperative versus postoperative ROM measurements; significance was defined as p<0.05.

ROM parameter	Preoperative (mean ± SD)	Postoperative (mean ± SD)	Mean difference	p-value
Flexion-extension (°)	81.5 ± 18.39	108.67 ± 12.17	27.17	<0.001
Adduction-abduction (°)	24.83 ± 11.48	43.17 ± 8.25	18.33	<0.001
Internal-external rotation (°)	10.83 ± 10.59	45.83 ± 8.52	35.00	<0.001

Functional outcomes

Functional outcomes at six months, assessed using the modified Harris Hip Score, are presented in Table 4. The mean HHS was 85.23 ± 8.99 (range: 63-93), with 43.33% (n=13) of patients achieving excellent scores (>90), 36.66% (n=11) good (80-89) scores, 6.66% (n=2) achieving fair (70-79) scores, and 13.33% (n=4) poor (<70) scores. The 80% good-to-excellent outcome rate is comparable to global benchmarks, indicating high surgical success in alleviating pain and improving function [15]. Notably, the patient with the retroverted cup initially had a poor HHS but achieved a good score post-revision, highlighting the correctability of malpositioning-related complications. However, Pearson’s correlation analysis revealed no significant associations between anteversion (r=0.12, p=0.52) or inclination (r=0.08, p=0.67) and HHS. This lack of correlation suggests that while cup positioning within the safe zone is crucial for stability, functional outcomes are influenced by multiple factors, including patient comorbidities, rehabilitation adherence, and surgical technique [16]. For instance, patients with poor HHS values often had confounding factors such as obesity or delayed physiotherapy, which may have limited functional recovery despite optimal cup placement. These findings emphasize the need for a holistic approach to THR, integrating precise surgical techniques with comprehensive postoperative care to maximize patient outcomes.

**Table 4:** Harris Hip Score outcomes HHS = Harris Hip Score HHS values were categorized as per standard criteria [12]. No p-values are reported as this table presents descriptive outcomes; Pearson’s correlation for cup orientation versus HHS is reported in the text (p>0.05).

HHS category	Score range	No. of patients	Percentage
Excellent	>90	13	43.33
Good	80-89	11	36.66
Fair	70-79	2	6.66
Poor	<70	4	13.33

Complications

Complications were minimal, with one dislocation (3.33%) attributed to the retroverted cup, and one leg length discrepancy (LLD) case (>3.2 cm, 3.33%). The mean LLD was 1.2 ± 0.8 cm, with no significant impact on HHS (p=0.45). The absence of infections reflects rigorous aseptic protocols, aligning with low infection rates in modern THR [12]. The dislocation case underscores the importance of intraoperative vigilance to prevent extreme malpositioning, as even a single error can lead to significant morbidity [17]. The LLD case, while not affecting HHS significantly, highlights the need for precise leg length restoration to optimize patient satisfaction and gait mechanics [14]. Overall, the low complication rate supports the reliability of the posterior approach when guided by anatomical landmarks, but the retroversion-related dislocation emphasizes the potential value of advanced imaging or navigation tools in high-risk cases.

## Discussion

This study assessed the impact of acetabular cup positioning on functional outcomes in 30 patients undergoing primary total hip replacement, revealing that 96.67% of cups achieved anteversion and 93.33% achieved inclination within the Lewinnek safe zone, as measured by postoperative CT scans [5]. These results align with prior research emphasizing the importance of precise intraoperative alignment to optimize joint stability and minimize complications such as dislocation [18]. The single case of retroversion (-5°) leading to posterior dislocation underscores the critical need to avoid extreme malpositioning, as retroverted cups disrupt the biomechanical balance, increasing impingement and instability risks [17,20]. In contrast, two patients with inclination angles slightly above the safe zone (51°, 57°) experienced no complications, suggesting that minor deviations in inclination may be less clinically significant than anteversion errors, a finding consistent with study results of Kennedy et al. [19]. The absence of a significant correlation between cup orientation and HHS (p>0.05) indicates that while proper alignment is essential for stability, functional outcomes are influenced by multifactorial elements, including patient comorbidities, rehabilitation adherence, and soft tissue management [12,14]. For instance, patients with poor HHS values often had confounding factors such as obesity or delayed physiotherapy, highlighting the need for comprehensive perioperative care to maximize outcomes [16].

The mean HHS of 85.23, with 80% of patients achieving good-to-excellent scores, is comparable to global benchmarks for THR, reflecting high surgical success in alleviating pain and restoring function [15]. Significant ROM improvements (p<0.001) across flexion-extension, abduction-adduction, and internal-external rotation further demonstrate the efficacy of THR in enhancing joint kinematics, particularly the 35° gain in rotational mobility, which is critical for daily activities like sitting and stair climbing [13]. These findings suggest that the posterior approach, guided by anatomical landmarks, is reliable for achieving optimal cup positioning in most cases, as evidenced by the low complication rate (3.33% dislocation, 3.33% significant LLD) [12]. However, the dislocation case emphasizes the limitations of manual techniques, as even experienced surgeons may encounter challenges in high-risk anatomies [17]. Advanced tools like intraoperative navigation and robotic assistance, which improve precision in cup placement, could reduce such risks; however, the cost and availability may limit their adoption in resource-constrained settings [3,10]. The absence of infections in our cohort reflects stringent aseptic protocols, aligning with modern THR standards, while the LLD case underscores the importance of meticulous leg length planning to optimize gait and patient satisfaction [14].

The Lewinnek safe zone, established in 1978, remains the gold standard for cup positioning, and yet recent studies question its universal applicability, citing patient-specific factors like spinopelvic alignment that may necessitate individualized targets [8,16]. Our study’s reliance on the safe zone was effective, but the retroversion-related dislocation suggests that dynamic factors, such as pelvic tilt during functional activities, warrant further exploration [14,21]. CT-based measurements provided precise postoperative validation, superior to X-rays for assessing anteversion, though challenges in obtaining high-resolution images highlight the need for robust imaging protocols [11].

Limitations of this study include the small sample size (n=30), which may limit the detection of subtle correlations between cup orientation and outcomes, and the short follow-up duration of six months, which may not capture long-term complications like polyethylene wear or aseptic loosening [22]. The HHS, while widely used, may have a ceiling effect, potentially masking nuanced differences in patient-reported outcomes [12]. Additionally, the study did not assess combined anteversion (acetabular and femoral components), which could influence stability and ROM [23]. Future research should involve larger, multicenter cohorts with extended follow-up to evaluate long-term outcomes and incorporate advanced imaging to assess spinopelvic parameters and combined anteversion. Integrating navigation technologies and patient-specific alignment protocols could further refine THR outcomes, particularly in complex cases [8].

## Conclusions

Precise acetabular cup positioning plays a critical role in achieving favorable clinical outcomes following total hip replacement. Our study demonstrated that the majority of patients with cup alignment within the Lewinnek safe zone experienced significant improvements in range of motion. However, no significant correlation was found between cup orientation and HHS (anteversion: r=0.12, p=0.52; inclination: r=0.08, p=0.67), indicating multifactorial influences on functional outcomes. Accurate measurement of cup orientation using computed tomography provides valuable postoperative validation, particularly in settings where intraoperative navigation is unavailable.

Based on our findings, we recommend heightened surgical attention to acetabular component alignment and the routine use of CT evaluation for postoperative assessment. Larger, multicenter studies with extended follow-up are warranted to confirm these results and further define best practices in total hip arthroplasty.
